# 4-Nitro­anilinium *p*-toluene­sulfonate

**DOI:** 10.1107/S1600536812040664

**Published:** 2012-09-29

**Authors:** P. K. Sivakumar, M. Krishnakumar, R. Kanagadurai, G. Chakkaravarthi, R. Mohankumar

**Affiliations:** a Department of Physics, MNM Jain Engineering College, Chennai 600 097, India; b Department of Physics, Presidency College, Chennai 600 005, India; c Department of Physics, CPCL Polytechnic College, Chennai 600 068, India

## Abstract

In the cation of the title salt, C_6_H_7_N_2_O_2_^+^·C_7_H_7_O_3_S^−^, the benzene ring makes a dihedral angle of 10.2 (2)° with the nitro group. In the crystal, the cations and anions are linked by weak N—H⋯O hydrogen bonds, forming a layer parallel to the *ac* plane. A weak C—H⋯O inter­action and π–π inter­actions [centroid–centroid distances of 3.738 (3) and 3.748 (3) Å] also observed within the layer.

## Related literature

For related structures of 4-toluene­sulfonate salts, see: Koshima *et al.* (2004 ▶); Biradha & Mahata (2005 ▶). For bond-length data, see: Allen *et al.* (1987 ▶).
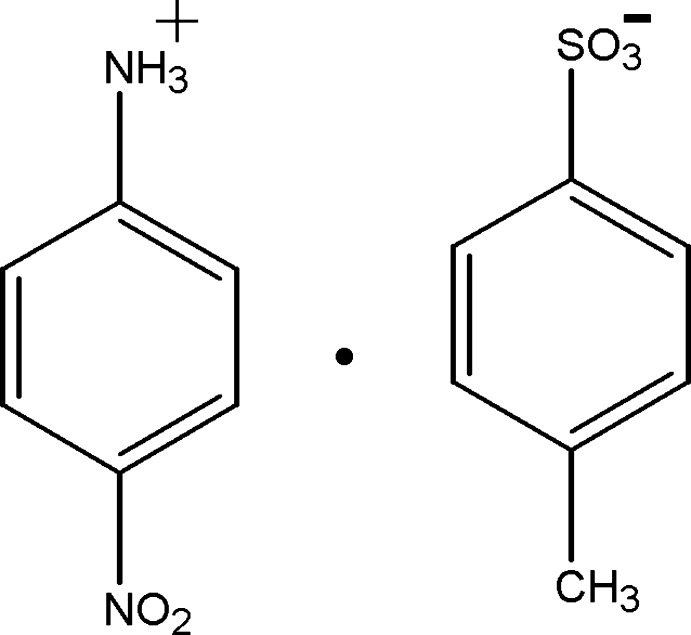


## Experimental

### 

#### Crystal data

C_6_H_7_N_2_O_2_^+^·C_7_H_7_O_3_S^−^
*M* *_r_* = 310.32
Monoclinic, 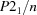

*a* = 6.216 (5) Å
*b* = 30.674 (4) Å
*c* = 7.405 (5) Å
β = 97.048 (5)°
*V* = 1401.2 (15) Å^3^
*Z* = 4
Mo *K*α radiation
μ = 0.26 mm^−1^
*T* = 295 K
0.30 × 0.24 × 0.20 mm

#### Data collection

Bruker Kappa APEXII diffractometer
Absorption correction: multi-scan (*SADABS*; Sheldrick, 1996 ▶) *T*_min_ = 0.928, *T*_max_ = 0.951
13942 measured reflections
3509 independent reflections
3232 reflections with *I* > 2σ(*I*)
*R*_int_ = 0.024

#### Refinement

*R*[*F* ^2^ > 2σ(*F* ^2^)] = 0.053
*wR*(*F* ^2^) = 0.134
*S* = 1.22
3509 reflections
192 parameters
H-atom parameters constrained
Δρ_max_ = 0.28 e Å^−3^
Δρ_min_ = −0.54 e Å^−3^

### 

Data collection: *APEX2* (Bruker, 2004 ▶); cell refinement: *SAINT* (Bruker, 2004 ▶); data reduction: *SAINT*; program(s) used to solve structure: *SHELXS97* (Sheldrick, 2008 ▶); program(s) used to refine structure: *SHELXL97* (Sheldrick, 2008 ▶); molecular graphics: *PLATON* (Spek, 2009 ▶); software used to prepare material for publication: *SHELXL97*.

## Supplementary Material

Crystal structure: contains datablock(s) global, I. DOI: 10.1107/S1600536812040664/is5199sup1.cif

Structure factors: contains datablock(s) I. DOI: 10.1107/S1600536812040664/is5199Isup2.hkl

Supplementary material file. DOI: 10.1107/S1600536812040664/is5199Isup3.cml

Additional supplementary materials:  crystallographic information; 3D view; checkCIF report

## Figures and Tables

**Table 1:** Hydrogen-bond geometry (Å, °)

*D*—H⋯*A*	*D*—H	H⋯*A*	*D*⋯*A*	*D*—H⋯*A*
N2—H14*B*⋯O3	0.89	2.09	2.856 (3)	144
N2—H14*A*⋯O2^i^	0.89	2.07	2.961 (3)	175
N2—H14*B*⋯O1^ii^	0.89	2.33	2.801 (3)	113
N2—H14*C*⋯O2^iii^	0.89	1.96	2.834 (3)	167
C12—H12⋯O3^iv^	0.93	2.59	3.193 (3)	123

Symmetry codes: (i) 
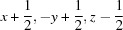
; (ii) 
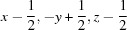
; (iii) 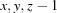; (iv) 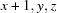.

**Fig. 1.**
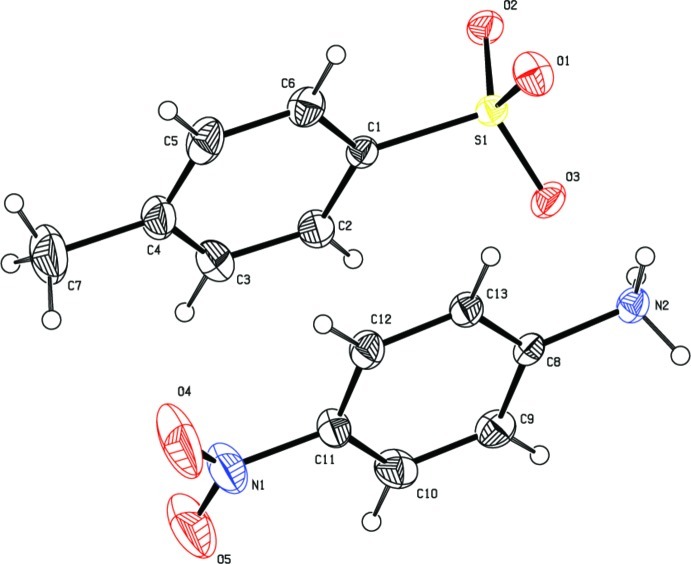
The molecular structure of the title compound, with atom labels and 30% probability displacement ellipsoids for non-H atoms.

**Fig. 2.**
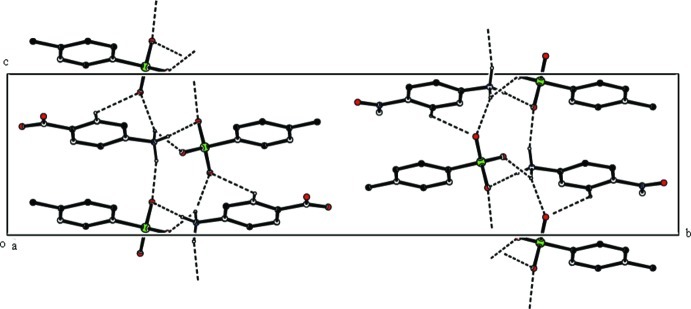
A packing diagram of the title compound, viewed down the a axis. Intermolecular hydrogen bonds are shown as dashed lines. H atoms not involved in hydrogen bonding have been omitted.

## supplementary crystallographic information

### Comment

The asymmetric unit of the title compound (Fig. 1) contains one C_6_H_7_N_2_O_2_^+^ cation and one C_7_H_7_O_3_S^-^ anion. The bond lengths and angles in both anion and cation are within normal range (Allen *et al.*, 1987) and those in the anion are comparable to those in other 4-toluenesulfonate salts (Koshima *et al.*, 2004; Biradha & Mahata, 2005). The crystal structure exhibit weak intermolecular N—H···O and C—H···O hydrogen bonds (Table 1 & Fig. 2) and π–π interactions. [*Cg*1···*Cg*2 (*x*, *y*, 1 + *z*) distance of 3.748 (3) Å; *Cg*2···*Cg*1 (*x*, *y*, -1 + *z*) distance of 3.748 (3) Å; *Cg*1···*Cg*2 (1/2 + *x*, 1/2 - *y*, 1/2 + *z*) distance of 3.738 (3) Å; *Cg*1 and *Cg*2 are the centroids of the rings (C1–C6) and (C8–C13), respectively.]

### Experimental

The title compound was formed from a mixture of 4-nitroaniline (2.15 g, 1 mmol) and p-toluenesulfonic acid (2.52 g, 1 mmol) in ethanol, which was stirred two hours at room temperature, giving a clear solution. After slow evaporation of ethanol for few days, single crystals suitable for X-ray diffraction were obtained.

### Refinement

H atoms were positioned geometrically and refined using riding model, with C—H = 0.93 Å and *U*_iso_(H) = 1.2*U*_eq_(C) for aromatic C—H, C—H = 0.96 Å and *U*_iso_(H) = 1.5*U*_eq_(C) for CH_3_, and N—H = 0.89 Å and *U*_iso_(H) = 1.5*U*_eq_(N).

### Crystal data

**Table:**

C_6_H_7_N_2_O_2_^+^·C_7_H_7_O_3_S^−^	*F*(000) = 648
*M**_r_* = 310.32	*D*_x_ = 1.471 Mg m^−^^3^
Monoclinic, *P*2_1_/*n*	Mo *K*α radiation, λ = 0.71073 Å
Hall symbol: -P 2yn	Cell parameters from 4052 reflections
*a* = 6.216 (5) Å	θ = 1.8–28.3°
*b* = 30.674 (4) Å	µ = 0.26 mm^−^^1^
*c* = 7.405 (5) Å	*T* = 295 K
β = 97.048 (5)°	Block, colourless
*V* = 1401.2 (15) Å^3^	0.30 × 0.24 × 0.20 mm
*Z* = 4	

### Data collection

**Table:**

Bruker Kappa APEXII diffractometer	3509 independent reflections
Radiation source: fine-focus sealed tube	3232 reflections with *I* > 2σ(*I*)
Graphite monochromator	*R*_int_ = 0.024
ω and φ scans	θ_max_ = 28.4°, θ_min_ = 1.3°
Absorption correction: multi-scan (*SADABS*; Sheldrick, 1996)	*h* = −8→8
*T*_min_ = 0.928, *T*_max_ = 0.951	*k* = −41→35
13942 measured reflections	*l* = −9→8

### Refinement

**Table:**

Refinement on *F*^2^	Primary atom site location: structure-invariant direct methods
Least-squares matrix: full	Secondary atom site location: difference Fourier map
*R*[*F*^2^ > 2σ(*F*^2^)] = 0.053	Hydrogen site location: inferred from neighbouring sites
*wR*(*F*^2^) = 0.134	H-atom parameters constrained
*S* = 1.22	*w* = 1/[σ^2^(*F*_o_^2^) + (0.0471*P*)^2^ + 0.984*P*] where *P* = (*F*_o_^2^ + 2*F*_c_^2^)/3
3509 reflections	(Δ/σ)_max_ < 0.001
192 parameters	Δρ_max_ = 0.28 e Å^−^^3^
0 restraints	Δρ_min_ = −0.54 e Å^−^^3^

### Special details

**Table:**

Geometry. All esds (except the esd in the dihedral angle between two l.s. planes) are estimated using the full covariance matrix. The cell esds are taken into account individually in the estimation of esds in distances, angles and torsion angles; correlations between esds in cell parameters are only used when they are defined by crystal symmetry. An approximate (isotropic) treatment of cell esds is used for estimating esds involving l.s. planes.
Refinement. Refinement of F^2^ against ALL reflections. The weighted R-factor wR and goodness of fit S are based on F^2^, conventional R-factors R are based on F, with F set to zero for negative F^2^. The threshold expression of F^2^ > 2sigma(F^2^) is used only for calculating R-factors(gt) etc. and is not relevant to the choice of reflections for refinement. R-factors based on F^2^ are statistically about twice as large as those based on F, and R- factors based on ALL data will be even larger.

### Fractional atomic coordinates and isotropic or equivalent isotropic displacement parameters (Å^2^)

**Table:**

	*x*	*y*	*z*	*U*_iso_*/*U*_eq_	
C1	0.4464 (3)	0.15687 (6)	1.0932 (3)	0.0291 (4)	
C2	0.3478 (4)	0.11695 (7)	1.0557 (3)	0.0413 (5)	
H2	0.2032	0.1156	1.0056	0.050*	
C3	0.4649 (5)	0.07893 (8)	1.0928 (4)	0.0526 (6)	
H3	0.3974	0.0522	1.0676	0.063*	
C4	0.6802 (5)	0.07991 (9)	1.1666 (4)	0.0513 (6)	
C5	0.7754 (4)	0.11997 (9)	1.2045 (4)	0.0508 (6)	
H5	0.9198	0.1212	1.2550	0.061*	
C6	0.6614 (3)	0.15854 (8)	1.1694 (3)	0.0410 (5)	
H6	0.7286	0.1852	1.1966	0.049*	
C7	0.8092 (6)	0.03827 (11)	1.2035 (5)	0.0829 (11)	
H7A	0.8506	0.0272	1.0914	0.124*	
H7B	0.7220	0.0170	1.2558	0.124*	
H7C	0.9369	0.0442	1.2866	0.124*	
C8	0.4640 (3)	0.17612 (6)	0.6035 (2)	0.0287 (4)	
C9	0.3447 (4)	0.13903 (8)	0.5530 (3)	0.0415 (5)	
H9	0.2015	0.1413	0.5002	0.050*	
C10	0.4402 (4)	0.09860 (8)	0.5817 (4)	0.0480 (6)	
H10	0.3627	0.0732	0.5504	0.058*	
C11	0.6539 (4)	0.09694 (7)	0.6584 (3)	0.0411 (5)	
C12	0.7739 (4)	0.13364 (7)	0.7077 (3)	0.0400 (5)	
H12	0.9177	0.1313	0.7590	0.048*	
C13	0.6768 (3)	0.17402 (7)	0.6797 (3)	0.0345 (4)	
H13	0.7543	0.1994	0.7118	0.041*	
N2	0.3582 (3)	0.21852 (6)	0.5788 (2)	0.0328 (4)	
H14A	0.4535	0.2395	0.6133	0.049*	
H14B	0.2490	0.2198	0.6459	0.049*	
H14C	0.3074	0.2221	0.4620	0.049*	
N1	0.7608 (5)	0.05441 (7)	0.6924 (4)	0.0669 (7)	
O1	0.4582 (3)	0.23868 (5)	1.0129 (3)	0.0461 (4)	
O2	0.1910 (3)	0.21560 (5)	1.20530 (19)	0.0384 (3)	
O3	0.1435 (2)	0.19798 (5)	0.8876 (2)	0.0379 (3)	
O4	0.9550 (4)	0.05402 (8)	0.7374 (5)	0.1119 (12)	
O5	0.6501 (5)	0.02194 (7)	0.6748 (5)	0.1009 (10)	
S1	0.30084 (7)	0.205912 (15)	1.04561 (6)	0.02737 (14)	

### Atomic displacement parameters (Å^2^)

**Table:**

	*U*^11^	*U*^22^	*U*^33^	*U*^12^	*U*^13^	*U*^23^
C1	0.0279 (9)	0.0332 (9)	0.0263 (8)	0.0032 (7)	0.0039 (7)	0.0001 (7)
C2	0.0390 (11)	0.0364 (11)	0.0463 (12)	−0.0012 (9)	−0.0033 (9)	−0.0008 (9)
C3	0.0632 (16)	0.0345 (11)	0.0586 (15)	0.0043 (11)	0.0014 (12)	−0.0016 (10)
C4	0.0590 (15)	0.0487 (14)	0.0465 (13)	0.0220 (11)	0.0077 (11)	0.0053 (11)
C5	0.0343 (11)	0.0656 (16)	0.0509 (14)	0.0147 (11)	−0.0004 (10)	0.0083 (12)
C6	0.0308 (10)	0.0471 (12)	0.0436 (12)	−0.0005 (9)	−0.0018 (9)	0.0015 (9)
C7	0.091 (3)	0.066 (2)	0.091 (3)	0.0408 (19)	0.009 (2)	0.0157 (18)
C8	0.0280 (9)	0.0348 (9)	0.0230 (8)	0.0016 (7)	0.0020 (7)	0.0026 (7)
C9	0.0308 (10)	0.0482 (12)	0.0434 (12)	−0.0059 (9)	−0.0039 (9)	−0.0025 (10)
C10	0.0478 (13)	0.0388 (12)	0.0562 (14)	−0.0112 (10)	0.0016 (11)	−0.0054 (10)
C11	0.0472 (12)	0.0325 (10)	0.0434 (12)	0.0047 (9)	0.0053 (10)	0.0030 (9)
C12	0.0336 (10)	0.0402 (11)	0.0444 (12)	0.0040 (8)	−0.0032 (9)	0.0009 (9)
C13	0.0300 (9)	0.0341 (10)	0.0374 (10)	−0.0007 (8)	−0.0041 (8)	−0.0003 (8)
N2	0.0288 (8)	0.0389 (9)	0.0303 (8)	0.0044 (7)	0.0026 (6)	0.0028 (7)
N1	0.0780 (18)	0.0361 (11)	0.0860 (19)	0.0084 (11)	0.0073 (14)	0.0039 (11)
O1	0.0391 (8)	0.0369 (8)	0.0628 (11)	−0.0085 (6)	0.0081 (7)	0.0031 (7)
O2	0.0421 (8)	0.0455 (8)	0.0283 (7)	0.0105 (6)	0.0072 (6)	−0.0024 (6)
O3	0.0338 (7)	0.0469 (8)	0.0307 (7)	0.0050 (6)	−0.0053 (6)	0.0016 (6)
O4	0.0718 (17)	0.0540 (14)	0.203 (4)	0.0273 (12)	−0.0121 (19)	0.0111 (17)
O5	0.107 (2)	0.0358 (11)	0.156 (3)	−0.0028 (12)	0.0016 (19)	0.0068 (14)
S1	0.0253 (2)	0.0303 (2)	0.0264 (2)	−0.00042 (16)	0.00236 (16)	−0.00106 (16)

### Geometric parameters (Å, º)

**Table:**

C1—C2	1.382 (3)	C9—C10	1.380 (3)
C1—C6	1.387 (3)	C9—H9	0.9300
C1—S1	1.768 (2)	C10—C11	1.380 (4)
C2—C3	1.384 (3)	C10—H10	0.9300
C2—H2	0.9300	C11—C12	1.375 (3)
C3—C4	1.383 (4)	C11—N1	1.472 (3)
C3—H3	0.9300	C12—C13	1.382 (3)
C4—C5	1.377 (4)	C12—H12	0.9300
C4—C7	1.515 (4)	C13—H13	0.9300
C5—C6	1.387 (3)	N2—H14A	0.8900
C5—H5	0.9300	N2—H14B	0.8900
C6—H6	0.9300	N2—H14C	0.8900
C7—H7A	0.9600	N1—O5	1.209 (3)
C7—H7B	0.9600	N1—O4	1.212 (4)
C7—H7C	0.9600	O1—S1	1.4436 (16)
C8—C13	1.374 (3)	O2—S1	1.4665 (16)
C8—C9	1.385 (3)	O3—S1	1.4507 (16)
C8—N2	1.459 (2)		
			
C2—C1—C6	119.73 (19)	C8—C9—H9	120.3
C2—C1—S1	120.68 (16)	C9—C10—C11	118.1 (2)
C6—C1—S1	119.59 (16)	C9—C10—H10	121.0
C1—C2—C3	119.8 (2)	C11—C10—H10	121.0
C1—C2—H2	120.1	C12—C11—C10	122.9 (2)
C3—C2—H2	120.1	C12—C11—N1	117.5 (2)
C4—C3—C2	121.3 (2)	C10—C11—N1	119.7 (2)
C4—C3—H3	119.3	C11—C12—C13	118.8 (2)
C2—C3—H3	119.3	C11—C12—H12	120.6
C5—C4—C3	118.1 (2)	C13—C12—H12	120.6
C5—C4—C7	120.7 (3)	C8—C13—C12	118.96 (19)
C3—C4—C7	121.2 (3)	C8—C13—H13	120.5
C4—C5—C6	121.7 (2)	C12—C13—H13	120.5
C4—C5—H5	119.1	C8—N2—H14A	109.5
C6—C5—H5	119.1	C8—N2—H14B	109.5
C1—C6—C5	119.3 (2)	H14A—N2—H14B	109.5
C1—C6—H6	120.3	C8—N2—H14C	109.5
C5—C6—H6	120.3	H14A—N2—H14C	109.5
C4—C7—H7A	109.5	H14B—N2—H14C	109.5
C4—C7—H7B	109.5	O5—N1—O4	123.8 (3)
H7A—C7—H7B	109.5	O5—N1—C11	118.2 (3)
C4—C7—H7C	109.5	O4—N1—C11	118.0 (2)
H7A—C7—H7C	109.5	O1—S1—O3	112.65 (11)
H7B—C7—H7C	109.5	O1—S1—O2	112.71 (10)
C13—C8—C9	121.95 (19)	O3—S1—O2	110.50 (11)
C13—C8—N2	119.34 (17)	O1—S1—C1	106.57 (10)
C9—C8—N2	118.70 (18)	O3—S1—C1	107.22 (9)
C10—C9—C8	119.4 (2)	O2—S1—C1	106.79 (9)
C10—C9—H9	120.3		
			
C6—C1—C2—C3	−0.6 (3)	C10—C11—C12—C13	0.0 (4)
S1—C1—C2—C3	179.55 (19)	N1—C11—C12—C13	−179.1 (2)
C1—C2—C3—C4	−0.2 (4)	C9—C8—C13—C12	−0.5 (3)
C2—C3—C4—C5	0.8 (4)	N2—C8—C13—C12	178.06 (18)
C2—C3—C4—C7	−178.8 (3)	C11—C12—C13—C8	0.0 (3)
C3—C4—C5—C6	−0.5 (4)	C12—C11—N1—O5	169.4 (3)
C7—C4—C5—C6	179.0 (3)	C10—C11—N1—O5	−9.8 (4)
C2—C1—C6—C5	0.8 (3)	C12—C11—N1—O4	−10.3 (4)
S1—C1—C6—C5	−179.28 (18)	C10—C11—N1—O4	170.6 (3)
C4—C5—C6—C1	−0.3 (4)	C2—C1—S1—O1	−152.38 (18)
C13—C8—C9—C10	0.9 (3)	C6—C1—S1—O1	27.7 (2)
N2—C8—C9—C10	−177.6 (2)	C2—C1—S1—O3	−31.5 (2)
C8—C9—C10—C11	−0.8 (4)	C6—C1—S1—O3	148.59 (17)
C9—C10—C11—C12	0.4 (4)	C2—C1—S1—O2	86.92 (19)
C9—C10—C11—N1	179.5 (2)	C6—C1—S1—O2	−92.96 (19)

### Hydrogen-bond geometry (Å, º)

**Table:**

*D*—H···*A*	*D*—H	H···*A*	*D*···*A*	*D*—H···*A*
N2—H14*B*···O3	0.89	2.09	2.856 (3)	144
N2—H14*A*···O2^i^	0.89	2.07	2.961 (3)	175
N2—H14*B*···O1^ii^	0.89	2.33	2.801 (3)	113
N2—H14*C*···O2^iii^	0.89	1.96	2.834 (3)	167
C12—H12···O3^iv^	0.93	2.59	3.193 (3)	123

Symmetry codes: (i) *x*+1/2, −*y*+1/2, *z*−1/2; (ii) *x*−1/2, −*y*+1/2, *z*−1/2; (iii) *x*, *y*, *z*−1; (iv) *x*+1, *y*, *z*.
